# Genomic Imprinting at the Porcine *DIRAS3* Locus

**DOI:** 10.3390/ani11051315

**Published:** 2021-05-03

**Authors:** Jinsoo Ahn, In-Sul Hwang, Mi-Ryung Park, Seongsoo Hwang, Kichoon Lee

**Affiliations:** 1 Functional Genomics Laboratory, Department of Animal Sciences, The Ohio State University, Columbus, OH 43210, USA; ahn.134@osu.edu; 2 Animal Biotechnology Division, National Institute of Animal Science, Rural Development Administration, Wanju 55365, Korea; insuri2642@korea.kr (I.-S.H.); mrpark45@korea.kr (M.-R.P.); hwangss@korea.kr (S.H.)

**Keywords:** DNA methylation, monoallelic expression, pigs, *DIRAS3*, whole-genome bisulfite sequencing, RNA-seq, hypothalamus

## Abstract

**Simple Summary:**

DNA methylation associated with one of the two alleles from parents is an important mechanism that causes a silencing of that allele, leading to expression of another allele only. There has been a lack of detailed studies on DNA methylation and expression patterns that are related to the *DIRAS3* gene in pigs. The objective of this study was to provide a comprehensive overview of DNA methylation and expression associated with the *DIRAS3* gene in pigs by generating an embryonic pig model and analyzing next-generation sequencing using pig embryos and adult pigs. Our results clearly showed the presence of DNA methylation near the *DIRAS3* gene in pigs and high expression of *DIRAS3* in the hypothalamus from adult pigs and expression of only one allele in all the tested tissues including the hypothalamus. In summary, our findings suggested DNA methylation might be related to those unique gene expression patterns during the development of pigs.

**Abstract:**

The epigenetic mechanisms underlying genomic imprinting include DNA methylation and monoallelic expression of genes in close proximity. Although genes imprinted in humans and mice have been widely characterized, there is a lack of detailed and comprehensive studies in livestock species including pigs. The purpose of this study was to investigate a detailed methylation status and parent-of-origin-specific gene expression within the genomic region containing an underexamined porcine *DIRAS3* locus. Through whole-genome bisulfite sequencing (WGBS) and RNA sequencing (RNA-seq) of porcine parthenogenetic embryos and analyses of public RNA-seq data from adult pigs, DNA methylation and monoallelic expression pattern were investigated. As a result, maternal hypermethylation at the *DIRAS3* locus and hypothalamus-specific and monoallelic expression of the *DIRAS3* gene were found in pigs. In conclusion, the findings from this study suggest that the presence of maternal hypermethylation, or imprints, might be maintained and related to monoallelic expression of *DIRAS3* during pig development.

## 1. Introduction

Genomic imprinting plays a crucial role in mammalian development and growth [1]. The epigenetic mechanisms underlying genomic imprinting include DNA methylation during mammalian embryonic development [2,3]. Differentially established DNA methylation in two parental germlines consists of epigenetic imprints and causes monoallelic expression of genes in close proximity [4]. For example, a differentially methylated region (DMR) at the *SGCE*/*PEG10* locus serves as the imprinting control region (ICR) which forces paternal allele-specific expression in humans [5,6], mice [7,8], pigs [9], and sheep [10]. Although genes imprinted in humans and mice have been widely characterized, there is a lack of detailed and comprehensive studies in livestock species including pigs. Our recent approach of the use of whole-genome bisulfite sequencing (WGBS) and RNA sequencing (RNA-seq) of porcine embryos that underwent parthenogenesis has facilitated verification of the imprinting status of known imprinted clusters and identification of novel imprinted genes [9,11]. This facilitation was attributed to a direct sequencing comparison between parthenogenetic and biparental porcine embryos. As a result, a detailed methylation status and parent-of-origin-specific gene expression within genomic regions containing underexamined target loci, such as the *DIRAS3* locus, could be further investigated. 

The distinct subgroup of the Ras family member 3 (*DIRAS3*) gene, also known as *ARHI* and *NOEY2*, encodes a small GTPase, which belongs to the Ras superfamily and serves as a tumor suppressor against various cancers including breast and ovarian cancers [11,12]. A maternal imprint and paternal monoallelic expression of the human *DIRAS3* gene have been reported to establish a functional allele that acts as a negative regulator of growth and development [13,14]. In pigs, although paternal expression of the *DIRAS3* gene at the fetal stage [15] and in adult skeletal muscles in which expression of *DIRAS3* is very low [16] was reported, DNA methylation imprints at the locus has not been investigated. In addition, according to the genotype-tissue expression (GTEx) project (https://gtexportal.org/home/gene/DIRAS3, accessed 15 January 2021), in an adult human, the *DIRAS3* gene was expressed the most in the hypothalamus among 54 sampled normal tissues and expressed in high levels in the nucleus accumbens (basal ganglia), ovary, and pituitary, whereas expressions in the other 50 normal human tissues were very low. However, detailed information about tissue-specific and/or monoallelic expression of the *DIRAS3* gene in adult pigs is still missing. Therefore, we explored DNA methylation using our parthenogenetic model and the expression of *DIRAS3* in the adult pig hypothalamus, as well as other major tissues (adipose, liver, lung, skeletal muscle, and spleen) by processing raw RNA-seq data from this study and from gene expression omnibus (GEO) series. 

Here, we aimed to determine concurrence of paternal allele expression and a maternal DMR within the promoter, exon, and intron regions of the porcine *DIRAS3* gene using RNA-seq and WGBS of parthenogenetic and biparental control embryos. In addition, tissue-specific and monoallelic expression of the *DIRAS3* gene in adult pigs was investigated by analyzing RNA-seq data. Our comprehensive analysis of DNA methylation and imprinted gene expression status of the porcine *DIRAS3* gene provides advanced groundwork for future studies of the *DIRAS3* locus.

## 2. Materials and Methods

### 2.1. Sample Collection and Data Used

Animal procedures were approved by the IACUC of the National Institute of Animal Science of Korea (approval number NIAS2015-670). Porcine oocytes were collected from 50 normal Landrace × Yorkshire × Duroc (LYD) pigs and matured in vitro and then parthenogenetic embryos were generated by electrical stimulation described in our previous reports [9,17]. After placing into oviducts of surrogate gilts, parthenogenetic embryos were developed until day 21. Furthermore, fertilized control porcine embryos were collected on day 21 after natural mating as described previously [9,17]. A publicly available dataset under accession number GSE158430 in the GEO database (https://www.ncbi.nlm.nih.gov/geo/) was accessed on 29 November 2020. Then, raw RNA-seq data for adipose tissue, hypothalamus, liver, lungs, skeletal muscles, and spleen of two 6-month-old adult male pigs (P348 and P350) (SubSeries GSE158412), raw H3K4me1 ChIP-seq data of the P348 hypothalamus (SubSeries GSE158427), and raw CTCF ChIP-seq data of the P350 hypothalamus (SubSeries GSE158416) were downloaded. We named P348—Pig 1, p350—Pig 2.

### 2.2. Whole-Genome Bisulfite Sequencing (WGBS) and RNA Sequencing (RNA-seq) 

For WGBS data generation, genomic DNA was isolated from the whole randomly collected control (CN, n = 3) and parthenote (PA, n = 3) embryos and processed as previously reported [9,17]. Briefly, an Accel-NGS Methyl-Seq DNA Library Kit (Swift Biosciences, Inc.; Ann Arbor, MI, USA) was used to optimize bisulfite conversion of genomic DNA according to the manufacturer’s instructions. PCR was conducted with adapter primers and Diastar™ EF-Taq DNA polymerase (Solgent, Daejeon, Korea) under the following thermal conditions: 3 min at 95 °C followed by 35 cycles of 30 s at 95 °C, 30 s at 60 °C, and 30 s at 72 °C, and a final extension for 5 min at 72 °C. After a bead-based clean-up, the PCR products were sequenced using a HiSeqX sequencer operated by Macrogen Inc. (Seoul, Korea) and 151 cycles for Read 1 and Read 2 were completed. The raw reads were checked for quality using FastQC (v0.11.7) (Babraham Bioinformatics, Cambridge, UK) and then trimmed and filtered out to remove adapters and reads shorter than 20 bp by using Trim Galore (v0.4.5) (Babraham Bioinformatics), leaving 846.5 (CN1), 862.1 (CN2), 866.5 (CN3), 839.7 (PA1), 856.9 (PA2), and 849.2 (PA3) million cleaned reads. Mapping to the pig reference genome (the Sscrofa11.1 assembly downloaded from https://www.ncbi.nlm.nih.gov/assembly/GCF_000003025.6, accessed 13 October 2020) and extracting the methylation ratio of every CpGs were conducted using BSMAP aligner (v2.87) (Baylor College of Medicine, Houston, TX, USA) [18]. 

To produce the transcriptome, RNA and cDNA library construction and RNA-seq were performed by TNT Research (Jeonju, Korea) as described [9,17], with total RNA isolated from the whole collected CN (*n* = 3) and PA (*n* = 3) embryos using the TRIzol reagent (Sigma-Aldrich, St Louis, MO, USA) following the manufacturer’s instructions. In brief, RNA samples treated with DNase I to avoid genomic DNA contamination were electrophoresed in 1.2% agarose gels to evaluate the integrity of RNA, which was then confirmed by the 28S/18S rRNA ratio (>2.0) and the RNA integrity number (RIN) (>7.0) using an Agilent 2100 BioAnalyzer. Using the ratios of A260/A280 and A260/A230 (1.8–2.0), the concentrations of RNA were assessed. One μg of total RNA was used to construct cDNA libraries with TruSeq RNA Sample Prep Kit v.2 (Illumina, San Diego, CA, USA). Quantification and qualification of the cDNA libraries were assessed by quantitative real-time PCR (qPCR) and using an Agilent 2100 Bioanalyzer, respectively. An Illumina HiSeq2500 RNA-seq platform was used to sequence the library products (101 nt paired-end). After quality checking, adapter trimming, and filtering by FastQC and Trim Galore, about 76.8 (CN1), 73.0 (CN2), 77.2 (CN3), 80.0 (PA1), 79.3 (PA2), and 80.3 (PA3) million cleaned reads were retrieved. STAR aligner (v.2.7.5) [19] was used to align the reads to the reference genome (Sscrofa11.1) with default parameter settings and produce BAM files of aligned reads. To normalize read coverages, deepTools (v3.5.0) [20] was used. 

### 2.3. Bioinformatics and Statistical Analysis 

All WGBS and RNA-seq alignments were visualized using the R/Bioconductor package Gviz (v1.32.0) [21] and/or Integrative Genomics Viewer (IGV) (v2.8.13) [22]. The downloaded raw RNA-seq and ChIP-seq data of adult pigs were analyzed to check tissue-specific and monoallelic expression. The downloaded reads were quality-checked by FastQC and trimmed using Trim Galore. STAR aligner [19] was used to map cleaned reads to the reference pig genome (Sscrofa11.1). Read coverages and alignments in those adult pigs were examined using BAM files and normalized using deepTools [20]. Differential expression was analyzed by quantifying the raw reads against the pig transcriptome using Salmon (v1.3.0) [23] and analyzing those quantified reads using the R/Bioconductor package DESeq2 (v.1.28.1) [24], and then the adjusted *p*-value < 0.05 was regarded as statistically significant. To call a DMR with the threshold of >10 CpGs, the maximum distance between CpGs < 300 bp, the mean methylation ratio difference (∆ ave) > 0.2 and the false discovery rate (FDR) < 0.05, metilene (v0.2-8) [25] was used. The shapiro.test function in the R package stats (v4.0.5) [26] was used to conduct the Shapiro–Wilk test to evaluate normality of the data. The Kruskal-Wallis test was performed using the kruskal.test function in the R package stats (v4.0.5) [26] to determine whether there is any significant difference between tissue groups regarding gene expression levels in adult pigs.

## 3. Results

### 3.1. Differential Expression and Methylation at the DIRAS3 Locus of Porcine Embryos

**Figure 1:**
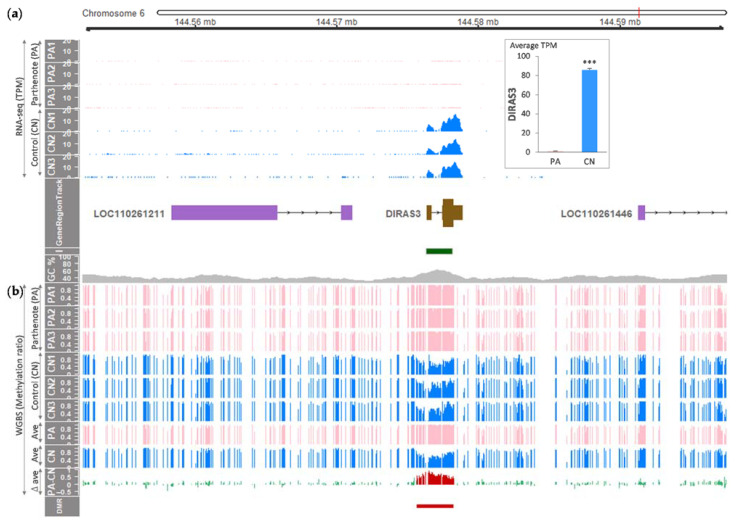
Profiling of the transcriptome and DNA methylome from the porcine parthenotes (PAs) and control embryos (CNs). (**a**) RNA-seq read coverages were normalized to transcripts per million (TPM). Differential expression (DE) of the porcine *DIRAS3* gene between PA and CN (*n* = 3 each) is shown in the box in the top-right corner (mean ± SEM; ***, adjusted *p* < 0.05). In GeneRegionTrack, purple boxes indicate exons of noncoding genes, and a protein-coding gene is shown as brown boxes with tall and short boxes indicating translated and untranslated regions, respectively. The direction of transcription is marked by horizontal arrows. I, CpG islands; *GC %*, GC content in percent. (**b**) CG methylation ratios of triplicates of PA and CN derived from whole-genome bisulfite sequencing (WGBS) are displayed, and averaged ratios were used to obtain differences in ratios (∆ ave, delta average). A differentially methylated region (DMR) between PA and CN (FDR < 0.05) is indicated with a red bar and overlaid on differentially methylated CGs in the PA-CN track with red histogram lines.

To verify paternal expression of the *DIRAS3* gene in day 21 porcine embryos, we compared *DIRAS3* expression between our model of parthenogenetic embryos without the paternal allele and biparental control embryos. By analyzing RNA-seq data, an exclusive expression of *DIRAS3* in the biparental control embryos with the paternal allele was found, but not in the parthenogenetic embryos (Figure 1a, Table 1). Surrounding noncoding genes (the porcine *LOC110261211* and *LOC110261446* genes encoding noncoding RNAs) were not expressed in both embryos. Furthermore, unlikely for humans, expression of an overlapping transcript (e.g., *GNG12-AS1* in humans) was not detected. Further differential gene expression analysis revealed that the expression of *DIRAS3* was more than 85-fold higher in the control embryos than in the parthenogenetic embryos (adjusted *p* < 0.05) (Figure 1a). In addition, our analysis of WGBS showed that a significantly higher DNA methylation at the *DIRAS3* locus occurred in the parthenogenetic embryos with two maternal alleles compared with the biparental control embryos (Figure 1b, Table 1, Table S1). This maternal hypermethylation established a DMR throughout the *DIRAS3* locus including a CpG island. Taken together, our combined results of RNA-seq and WGBS provided strong evidence of maternal imprints that give rise to a paternal expression of the porcine *DIRAS3* gene.

### 3.2. Sequence Elements and Differentially Methylated CpGs within the Porcine DIRAS3 Locus

**Figure 2:**
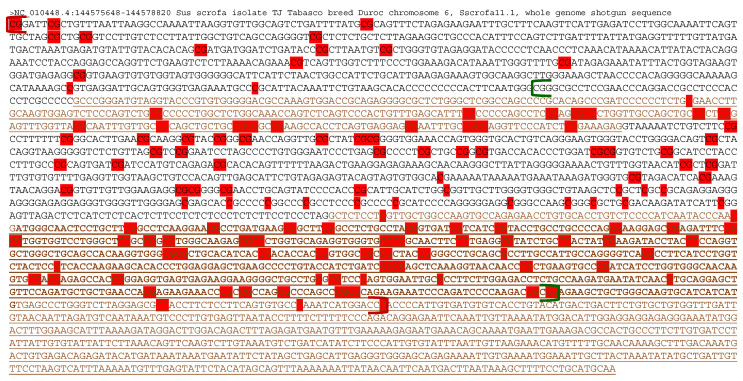
Distribution of differential methylation (i.e., maternal hypermethylation) in the *DIRAS3* locus of the porcine parthenogenetic (PA) embryos compared to the control (CN) embryos. The nucleotide sequence of the genomic DNA covers a promoter (black), exon 1 (brown, underlined), intron (black), and exon 2 (brown, underlined) of the porcine *DIRAS3* gene from the beginning to the end. Brown bold faces in exon 2 indicate a translated region. This partial genomic DNA sequence in chromosome 6 (chr6 or NC_010448.4:144575648-144578820) of the reference pig genome (Sscrofa11.1 or susScr11) was retrieved from the NCBI Nucleotide database (https://www.ncbi.nlm.nih.gov/nuccore, accessed 15 January 2021). The start and end of the CpG island (chr6:144576300-144578127) and DMR (displayed in Figure 1) are denoted with green and red brackets, respectively. Differentially methylated CpGs (or Cs) between PA and CN (i.e., hypermethylated in PA) are highlighted with red. Highlighted Gs indicate methylated Cs on the minus strand (coding strand). To call a DMR with a threshold, metilene was used, as mentioned in the Materials and Methods.

Since DNA hypermethylation has been recognized as an epigenetic regulator of gene expression that potentially silences or downregulates tumor suppressor genes such as *DIRAS3* [27], detailed maternal hypermethylation status was profiled in the porcine *DIRAS3* locus. Unlike humans in whom CpG islands are separated into three pieces at the promoters and the gene body, the *DIRAS3* locus in pigs contains one consecutive CpG island spanning between a part of the promoter region and a large portion of the gene body (exon 1, intron, and exon 2) (Figure 2). Because differentially methylated CpGs (or Cs) were distributed throughout the CpG island and even farther in the promoter and exon 2, a DMR was also consecutive throughout the porcine *DIRAS3* locus. The consecutiveness was detected even with conservative criteria of at least ten differentially methylated CpGs with the maximum distance between CpGs of less than 300 bp, which was changed from the default maximum distance of 500 bp in the DMR caller, metilene (Figure 2). On the other hand, in humans, three separate DMRs that corresponded to three CpG islands were reported [28]. In sum, in pig embryos, maternal hypermethylation occurred throughout the promoter and the gene body of the *DIRAS3* gene beyond the CpG island, establishing one DMR.

### 3.3. Tissue-Specific and Monoallelic Expression of the DIRAS3 Gene in Adult Pigs

**Figure 3:**
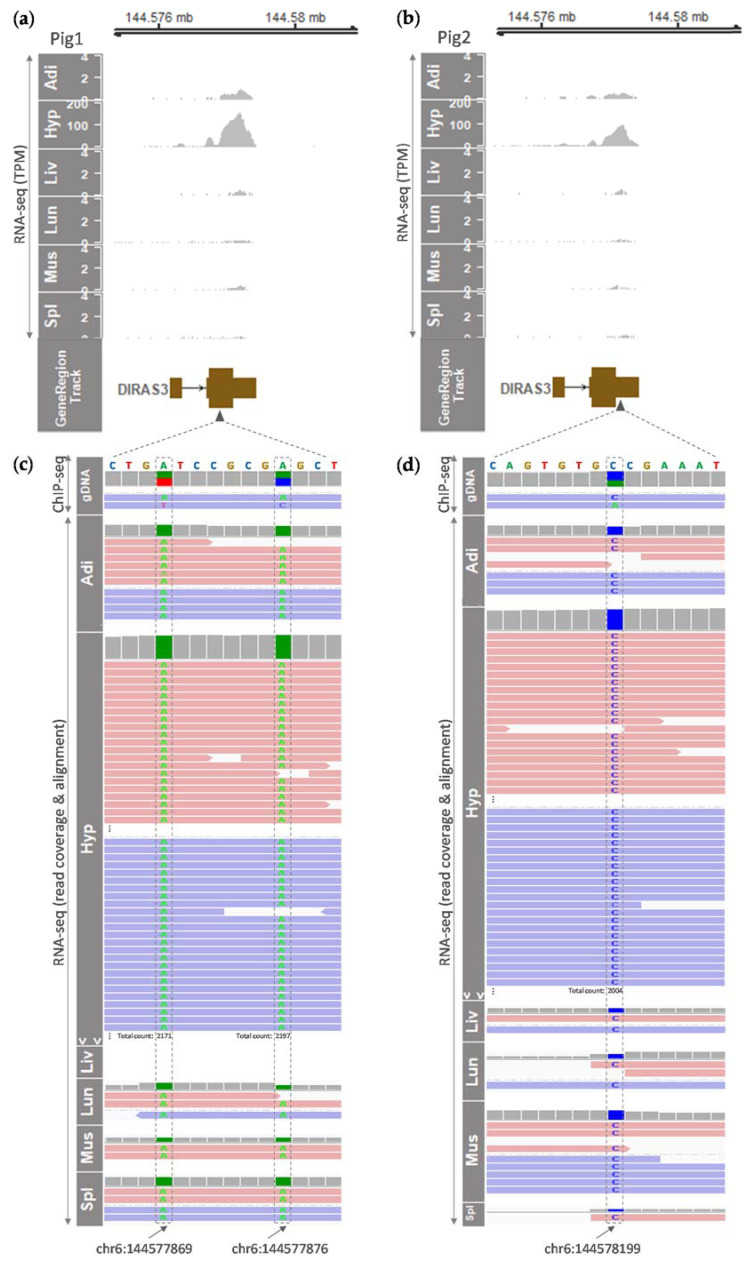
Tissue-specific and monoallelic expression of the *DIRAS3* gene in adult pigs (six-month-old). Expressions of the *DIRAS3* gene in various tissues from pig 1 (**a**) and pig 2 (**b**) were plotted based on TPM values obtained from RNA-seq. In addition, monoallelic expressions of the *DIRAS3* gene in those various tissues were analyzed using read coverages and alignments in BAM files from RNA-seq as follows: for pig 1 (**c**), expression of reference alleles at two heterozygous sites (A on chr6:144577869; A on chr6:144577876) instead of alternative alleles (T and C, respectively) is shown, and for pig 2 (**d**), expression of a reference allele at a heterozygous site (C on chr6:144578199) instead of an alternative allele (A) is displayed. Raw RNA-seq and ChIP-seq data were retrieved from GSE158430 and processed as described in the Materials and Methods. Adi, adipose tissue; gDNA, genomic DNA; Hyp, hypothalamus (brain); Liv, liver; Lun, lung; Mus, skeletal muscle; Spl, spleen. More reads were only present in the Hyp as indicated. Black arrowheads at the second exon of the *DIRAS3* transcript denote locations of nucleotide sequences in (**c**,**d**). Black arrows at the bottom indicate genomic coordinates (chromosome and base position) of heterozygous sites.

We further examined the expression of *DIRAS3* in adult pigs to show whether imprinted gene expression maintains at the adult stage. First, we explored whether tissue-specific expression patterns exist. Considering that the *DIRAS3* gene in humans was expressed predominantly in the brain (hypothalamus, nucleus accumbens), as well as in the ovary and the pituitary (as mentioned above), we predicted that this expression pattern may be conserved in pigs. Our analysis of RNA-seq data from two six-month-old male pigs revealed the *DIRAS3* gene expression appeared to be hypothalamus-specific among the analyzed major pig tissues (Table 2, Figure 3a,b). However, tissue distribution was non-normal according to the Shapiro–Wilk test, and therefore a non-parametric test (the Kruskal–Wallis test) was performed. The result showed that mRNA expressions of both *LOC110261211* and *DIRAS3* were not statistically different between the analyzed tissues (Table 2). On the other hand, these TPM values from the hypothalamus were approximately five to eight times higher than those values from the control embryos in Figure 1a. Among other tissues including adipose tissue, liver, lung, skeletal muscle, and spleen, *DIRAS3* expression was observed as slightly higher in adipose tissue, but the absolute expression level was low (Figure 3a,b). Secondly, further analysis of read coverages and alignments showed that only reference alleles (A alleles in pig 1 and C allele in pig 2) were expressed at heterozygous sites in all the tested tissues instead of the alternative alleles shown in published ChIP-seq data from genomic DNA (gDNA) of the same pigs, indicating monoallelic expression of the *DIRAS3* gene (Figure 3c,d). Overall, these data suggest that the imprinted expression of *DIRAS3* is maintained at the adult stage in which *DIRAS3* expression shows a tendency of hypothalamus-enriched expression.

## 4. Discussion

Our findings in this study suggest that the *DIRAS3* gene in porcine embryos is subjected to silencing of its promoter region in the maternal allele through DNA hypermethylation, which is one of the genomic imprinting mechanisms including direct silencing of promoters, inhibition of long-range communication between promoters and enhancers, repression of long-noncoding RNA (lncRNA), isoform-dependent silencing, and histone modifications [29,30,31]. This direct silencing via maternal DNA hypermethylation might lead to paternal allele-specific expression of *DIRAS3* in biparental control embryos, but not in parthenogenetic embryos, where it is deficient in the paternal allele. As such, our generation of parthenogenetic embryos enabled detection of paternal expression by all-or-none fashion (i.e., all biparental controls having a paternal allele, and no parthenotes without any paternal allele), along with a construction of the methylome and the transcriptome. 

Further analysis of DNA sequence elements and methylated CpGs showed that both the regions of CpG island and maternal methylation were more widely spread in pigs than in humans. Since aberrant DNA methylation on the paternal allele has been identified as a major inhibitor of the imprinted expression of *DIRAS3* [14], a detailed overview of methylated regions is needed. It was reported that in human patients with breast cancer and ovarian cancer, hypermethylation in both CpG islands, I and II, was associated with reduced expression of *DIRAS3* [32,33]. This CpG island II is located at the proximal promoter and exon 1 of the human *DIRAS3* isoform [28,32] and corresponds, in part, to the pig CpG island, suggesting paternal hypermethylation in this region could be related to reduced *DIRAS3* expression. Given that the porcine DMR was extended 652 bp upstream and 86 bp downstream beyond the CpG island (Figure 2), there might be additional gene regulation via DNA methylation.

Furthermore, our data analysis revealed that in adult male pigs, the expression of the *DIRAS3* gene tended to be hypothalamus-specific and monoallelic, suggesting maintenance of the imprinted gene expression during development. Despite the tendency of hypothalamus specificity, non-normal distribution and non-statistical significance (*p* = 0.08204) of the distribution of *DIRAS3* might be due to the low sample size. Considering tissue-specific *DIRAS3* expression patterns shown in the GTEx project (predominant in the human hypothalamus, nucleus accumbens, ovary, and pituitary) and given the hypothalamus-specific tendency in the current study, the reason why a previous study reported ubiquitous expression of porcine *DIRAS3* in tested tissues [16] might be due to saturation of RT-PCR. There are also conflicting reports on the imprinted status of *DIRAS3* in pig placenta, as one report mentioned a lack of imprinted isoform in the placenta [15], while another report displayed monoallelic expression of *DIRAS3* in the placenta [34]. Based on our plot of RNA-seq read coverages, there was only one expressed transcript in the *DIRAS3* locus, unlike humans in whom a longer isoform exists according to the Ensembl database (http://www.ensembl.org/, accessed 15 January 2021). To clarify the imprinted status of one *DIRAS3* transcript in the pig placenta, future studies will need to investigate both monoallelic expression and methylation status and link them to provide comprehensive profiling. 

Overall, the porcine *DIRAS3* locus has similarities and differences compared to the human ortholog. The presence of maternal hypermethylation (imprints), tissue-specific expression tendency in the hypothalamus, and its monoallelic expression pattern were similar. Differences covered the locations and numbers of the CpG island and DMR, as well as non-existence of an overlapping *GNG12-AS* transcript in pigs. Further investigation across species in a comparative manner will improve our understanding on the underlying genetic and epigenetic mechanisms related to the *DIRAS3* locus.

## 5. Conclusions

Although genes imprinted in humans and mice have been widely characterized, including tumor suppressor genes such as *DIRAS3* or *ARHI*, there is a lack of detailed and comprehensive studies in livestock species including pigs. The findings from this study suggest that the presence of maternal hypermethylation, or imprints, might be maintained and related to monoallelic expression of *DIRAS3* during pig development. Our approach based on parthenogenesis and various next-generation sequencing data promotes comprehensive and detailed identification of imprinted genes and loci in a genomic context.

## Figures and Tables

**Table 1:** DNA methylation and gene expression in PA and CN embryos.

Gene/Locus	Coordinate (chr6) Start & End	Embryo	Methylation (Ratio, ave)	Expression (TPM, ave)	*p*-Value	Adjusted *p*-Value
*LOC110261211*	144,558,280 144,571,021 (12,741 bp)	PA	0.88 ± 0.01	0.16 ± 0.00	0.69	0.89
		CN	0.86 ± 0.01	0.18 ± 0.05		
DMR	144,575,647 144,578,475 (2828 bp)	PA	0.95 ± 0.00	NA	NA	NA
		CN	0.50 ± 0.01	NA		
*DIRAS3*	144,576,342 144,578,820 (2478 bp)	PA	0.96 ± 0.00	1.00 ± 0.17	<0.05	<0.05
		CN	0.49 ± 0.01	85.78 ± 1.55		
*LOC110261446*	144,591,277 144,721,840 (130,563 bp)	PA	0.81 ± 0.00	0.00 ± 0.00	NA	NA
		CN	0.77 ± 0.00	0.00 ± 0.00		

PA, parthenogenetic embryo; CN, control embryo. Values are presented as average (ave) ± SEM. The p-values and adjusted p-values were obtained from DESeq2 for detecting differentially expressed genes. NA, not available.

**Table 2:** Gene expression in various tissues of adult pigs.

Gene	Expression (TPM, ave)					
	Adipose Tissue	Hypothalamus	Liver	Lung	Muscle	Spleen
*LOC110261211*	0.07 ± 0.03	0.17 ± 0.09	0.05 ± 0.01	0.12 ± 0.01	0.02 ± 0.00	0.11 ± 0.02
*DIRAS3*	1.60 ± 0.30	468.30 ± 160.39	0.26 ± 0.04	0.57 ± 0.06	0.34 ± 0.09	0.31 ± 0.06
*LOC110261446*	0.00 ± 0.00	0.00 ± 0.00	0.00 ± 0.00	0.00 ± 0.00	0.00 ± 0.00	0.00 ± 0.00

For both *LOC110261211 and DIRAS3,* the distribution significantly differed from normal distribution according to the Shapiro–Wilk test (*p*-values were 0.0423 and 1.267 × 10^−5^, respectively). The *p*-values from the Kruskal–Wallis test were 0.148 (*LOC110261211*) and 0.08204 (*DIRAS3*), which are lower than the significance level (0.05) indicating that there were no significant differences between the tissue groups.

## Data Availability

Data supporting the results were downloaded from GSE158430 and can be found within the Supplementary Materials.

## Supplementary Materials

The following are available online at https://www.mdpi.com/article/10.3390/ani11051315/s1, Table S1: Differentially methylated region (DMR) called by the metilene software.
